# Our First Line of Defence

**Published:** 1901-08-24

**Authors:** 


### Our First Line of Defence.

The greatest enemy we have, who never ceases in his attacks and must be met at all times and in all places, is insanitation. To combat and defeat this foe is the first duty of all Governments, and the history of the last quarter of the nineteenth century will always stand out as the epoch when serious endeavours were first made to organise and administer on some rational basis the laws relating to public health. The gradual growth of our Constitution, of which we are so proud, chiefly because it has been a spontaneous and natural outcome of the needs of the people and has never been recast by revolutionary and theoretical doctrinarians, has nevertheless led to many inconvenient and complicated systems. Thus the management of local affairs in the early eighties was in a state of absolute chaos, chiefly caused by the enormous multiplication of bodies or boards each of which undertook some special duty in some special district. The result was that the whole country was divided into Parishes Ecclesiastical and otherwise, Poor Law and Highway Parishes, Lighting and Watching Districts, Improvement Act Districts, Urban, Rural, and Port Sanitary Districts, Burial Board Districts, School Board Districts, Unions and many others, all of which were distinct areas administered by distinct bodies, so that in 1883 the total number of local authorities who taxed the ratepayer was 27,000 and they taxed him by eighteen different kinds of rates. Since then successive Governments have amalgamated these various bodies into a few of which the County and District Councils chiefly concern us at the present moment.

These bodies have important powers given to them but are under the ultimate control of the Local Government Board, whose president is a minister responsible to the King and Parliament. Of these one of the chief is the appointment of Medical Officers who act as expert advisers on all questions affecting the health of the country.

But although much has been done to simplify the methods of local government, the discussion opened by Dr. Francis Bond at the British Medical Association shows that we are still far from reaching a condition wherein the greatest efficiency and economy are combined.

It seems to us that the chief desideratum is a medical officer appointed in each county or in some other convenient defined area who shall be remunerated sufficiently to enable him to devote his whole time to his public duty. Sixty-two authorities are empowered to appoint a County Medical Officer of Health, but only 14 have appointed a whole-time medical officer, and seven more an officer for special purposes, while 41 have no officer. This fact shows conclusively that the councils are not doing their duty. A laboratory for water, food, sewage, and other analyses and bacteriological examinations should also be instituted in each county or area under the direct superintendence of this officer. It is manifestly impossible for each District Council to afford the whole services of a medical man, but it is desirable that competent practitioners should be appointed for areas not larger than they can easily keep a constant watch upon, while all special work and investigations should be relegated to the central authority.

The whole science of public health is in its infancy, and although rapidly advancing is still largely experimental. It is therefore desirable that local authorities should be permitted to adopt various methods of isolation, notification, hospital arrangements, and sewage and refuse disposal, always assuming that in the opinion of those best qualified to judge no radical defect exists in the general principle. In these matters we are still far from unanimous, and it is, therefore, a great mistake to encourage any tendency to crystallise methods by too great a supervision by the Local Government Board. With finance, uniformity is absolutely essential, and this is ensured by the Government auditors, but in actual administration as little check as possible should be placed on the local authorities.

As the law exists at present the County Councils and the Local Government Board have powers of creating Urban Districts, and if the Parish or District Council refuses to act, it can do the work itself. The duties relating to public health are laid down in great detail in the Public Health Act of 1875, and Amending Acts, and there is no doubt these powers are already very great but are not absolute. But no sanitary authority can be expected to be in a position to initiate and carry to success any great sanitary measure unless it is advised by a competent expert and has such control of its own local affairs as is consistent with the welfare of the whole community.

